# Diagnostic pathways of Chagas disease in Spain: a qualitative study

**DOI:** 10.1186/s12889-022-14938-4

**Published:** 2023-02-14

**Authors:** Laura Iglesias-Rus, Teresa Boquete, María Romay-Barja, Agustín Benito, Briggitte Jordan, Teresa Blasco-Hernández

**Affiliations:** 1 National Centre for Tropical Medicine, Carlos III Institute of Health, Madrid, Spain; 2 Biomedical Research Networking Centre in Infectious Diseases, CIBERINFEC, Madrid, Spain; 3 Fundación Mundo Sano, Madrid, Spain

**Keywords:** Chagas disease, Neglected diseases, Health behaviour, Health services needs and demand

## Abstract

**Background:**

Due to the mobility of the population in recent years and the spread of Chagas disease (CD) to non-endemic regions, early diagnosis and treatment of CD has become increasingly relevant in non-endemic countries. In order for screening to be effective, health system accessibility must be taken into consideration. This study uses Tanahashi’s Health Service Coverage model to gain a deeper understanding of the main diagnostic pathways for Chagas disease in a non-endemic country and the barriers and bottlenecks present in each pathway.

**Methods:**

This study used a qualitative design with a phenomenological approach. Twenty-one interviews, two focus group sessions, and two triangular group sessions were conducted between 2015 and 2018 with 37 Bolivian men and women diagnosed with CD in Madrid, Spain. A topic guide was designed to ensure that the interviewers obtained the data concerning knowledge of CD (transmission, symptoms, and treatment), attitudes towards CD, and health behaviour (practices in relation to CD). All interviews, focus groups and triangular groups were recorded and transcribed. A thematic, inductive analysis based on Grounded Theory was performed by two researchers.

**Results:**

Three main pathways to CD diagnosis were identified: 1) pregnancy or blood/organ donation, with no bottlenecks in effective coverage; 2) an individual actively seeking CD testing, with bottlenecks relating to administrative, physical, and time-related accessibility, and effectiveness based on the healthcare professional’s knowledge of CD; 3) an individual not actively seeking CD testing, who expresses psychological discomfort or embarrassment about visiting a physician, with a low perception of risk, afraid of stigma, and testing positive, and with little confidence in physicians’ knowledge of CD.

**Conclusions:**

Existing bottlenecks in the three main diagnostic pathways for CD are less prevalent during pregnancy and blood donation, but are more prevalent in individuals who do not voluntarily seek serological testing for CD. Future screening protocols will need to take these bottlenecks into consideration to achieve effective coverage.

## Background

Chagas disease (CD) is a chronic parasitic infection caused by *Trypanosoma cruzi* (*T. cruzi*). Triatomine bugs are the vectors in endemic countries, but blood transfusions, solid organ transplants, and particularly congenital transmission are also potential routes in endemic and non-endemic regions alike [1].

The acute phase of Chagas disease lasts 4-8 weeks, with detectable parasitaemia and no symptoms, followed mainly by an indeterminate chronic phase that remains asymptomatic, without signs or symptoms of visceral involvement [1]. Between 30% and 40% of patients will develop megaviscera and/or cardiomyopathy if CD is left untreated, costing health systems worldwide $627 million per year and 806,170 disability-adjusted life years [2].

The available drugs for the treatment of CD are benznidazole and nifurtimox, and their use in the acute phase and in recently infected individuals is highly effective [1, 3]. In addition, 100% of children with congenital CD are cured if treated during their first year of life, and treatment of infected women before pregnancy prevents vertical transmission [1, 3–6]. However, its effectiveness decreases in the indeterminate chronic phase or if visceral involvement is present, meaning that only early diagnosis prevents cardiac and gastrointestinal damage [1, 3, 7].

CD is endemic to 21 countries in Latin America, especially in rural settings [1]. Its spread to non-endemic regions, such as North America and Europe, is the result of increased migration in recent decades [1]. Prevalence among Latin American migrants living in Europe is approximately 4.2%, with Bolivian migrants bearing the brunt of the infections (18.1%) [8]. It is estimated that there are between 48,000 and 86,000 cases of CD in Spain, but only 10% of them have been diagnosed [9]. About 81% of cases in Spain have been reported among the Bolivian population, and a number of studies indicate that Madrid has a prevalence rate of 27.7% among Bolivians [10, 11].

Screening for CD in the Latin American population is a cost-effective strategy in non-endemic areas, even if CD is not officially present [7, 12–14]. Several European countries have passed legislation to ensure blood transfusions and organ transplants are screened, but screening of the at-risk population has not been fully implemented in Spain despite the high number of migrants from Latin America [7, 12]. However, a number of Spanish regions and healthcare professionals have designed protocols and guidelines for testing migrants from endemic areas [14, 15]. In addition, the implementation of a screening must take into consideration access to the healthcare system. Healthcare accessibility is the degree to which individuals are helped or hindered in accessing and receiving care and services from the health system [16], which is a complex situation influenced by a wide range of factors. In fact, many authors have developed theoretical models to explain this concept [17].

**Fig. 1:**
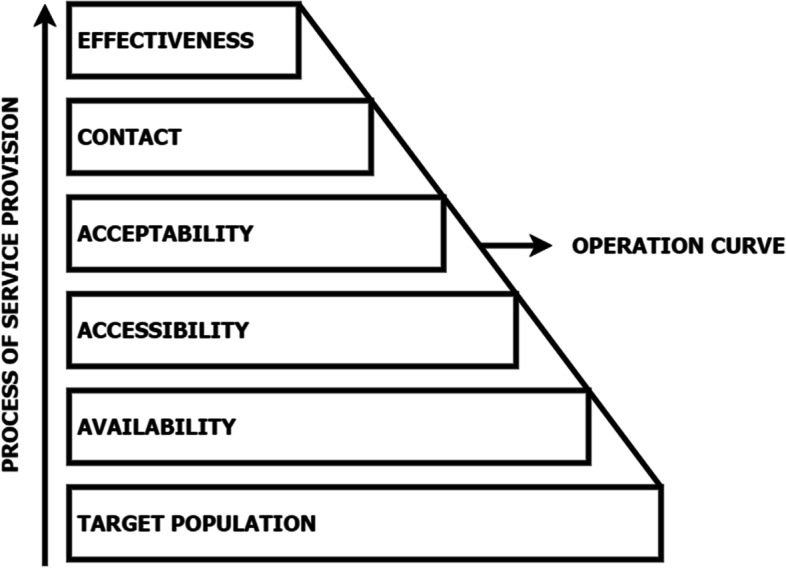
Diagram illustrating the relationships between service provision and coverage [18]

Tanahashi defines health service coverage as the ability of a health service to successfully interact with the target population [18]. This process depends on the following factors: availability, accessibility, acceptability, contact, and effectiveness [18]. In order to provide a service, it is necessary to: provide manpower, infrastructure, and supplies (availability); locate them within a reasonable reach (accessibility); make them acceptable to the intended users in line with their social and cultural norms (acceptability); measure the actual degree of contact with the health service (contact); and ascertain whether the service has successfully met healthcare needs (effectiveness) [18] (Fig. 1).

Tanahashi’s model also identifies whether the service has a problem or bottleneck when there is a significant difference between these factors. This model is currently used for quantitative and qualitative analysis of access to health services in vulnerable populations. Some of the bottlenecks identified have been linked to culturally inappropriate services, knowledge about the health services available, staff shortages, lack of residence permits, service providers’ practice, lack of trust in the service, and professionals without specialised training [18–23]. However, to the best of our knowledge, no studies have been found applying Tanahashi’s model to CD testing. Therefore, a thorough understanding of the pathways that enable CD diagnosis in the Bolivian population and its bottlenecks may provide valuable insight that could help refine or adapt interventions to improve health services in non-endemic countries [1]. This study assessed the current pathways to CD diagnosis in Madrid, Spain, and their bottlenecks based on Tanahashi’s Health Service Coverage model.

## Methods

### Setting

The study was conducted in Madrid, Spain, a country in which CD is non-endemic. The January 2017 municipal census reported a total of 15,951 Bolivians living in Madrid, of whom 9,193 were women and 6,758 were men. This population is mainly distributed among the Usera, Carabanchel, Puente de Vallecas, and Latina districts in Madrid [10].

Healthcare in Spain is provided at two levels (hospitals and primary care facilities), and serological testing for CD is currently available at both levels. Current legislation in Madrid allows for treatment at any hospital or primary care facility [24]. Access to healthcare in Spain was free of charge until 2012, when it was linked to social security registration or limited to emergency services for undocumented migrants [25]. Legislation changed again in 2018, making it mandatory for migrants to prove a minimum three-month stay in Spain in order to be able to access the public health system [26]. Despite these limitations, a number of Spanish hospitals and primary care facilities managed to diagnose and treat this population.

### Study design

This study used a qualitative research design with a phenomenological approach consisting of semi-structured interviews, focus group sessions (FGSs), and triangular group sessions (TGSs), which were the main data collection techniques. The triangular group technique merges individual and group discourses with a less structured format and fewer participants [27, 28]. 

This study is part of a project assessing access to and use of healthcare services among the Bolivian population in Madrid. A questionnaire was employed to explore the Bolivian population’s knowledge, attitudes, and practices regarding CD, care itineraries, screening, management, and treatment of CD [10]. This study presents the results of interviews, FGSs and TGSs conducted with the Bolivian population regarding CD diagnostic pathways and associated bottlenecks.

### Participant recruitment

Based on the types of sampling methods described by Teddlie and Yu [29], this study used a convenience sampling technique. Inclusion criteria for participants were the following: resident in Madrid and over 18 years of age. The sample was stratified into Bolivian men and women who had tested positive for CD because migrants from Bolivia have the highest prevalence of CD in Europe [8].

The research team contacted participants through the Bolivian Consulate and through a community health agent considering their time availability on weekdays and weekends. Recruitment of participants was carried out until saturation was reached (i.e. until no relevant information was obtained from additional interviews) [30].

### Data collection

Between 2015 and 2016, FGSs, TGSs, and individual interviews were conducted with women, and between 2017 and 2018, seven individual interviews were carried out with men; FGSs with men were not conducted because they did not come to meetings.

A topic guide was designed for the interviews, FGSs and TGSs to ensure that interviewers obtained the data needed to meet the study objectives and explore emerging themes in greater depth. The topic guide was reviewed by two researchers to ensure their comprehensibility and potential to boost discussion following the Kvale model [31]. The guide was modified during data collection with the subsequent transcriptions of the FGSs, TGSs, and interviews when new themes emerged that required the inclusion of new questions or greater detail.

The topic guide included knowledge of CD (transmission, symptoms, and treatment), attitudes towards CD, and health behaviour (practices in relation to CD). Interviews, FGSs and TGSs were conducted in Spanish; each interview lasted 45 min on average, and FGSs and TGSs lasted 60-90 min.

Digital voice recorders were used for all data collection techniques, and sociodemographic variables (sex, age, region of origin, level of education, employment status, public health coverage, number of children, and years residing in Spain) were gathered as supplementary information. These data were entered into a spreadsheet and were attributed codes to ensure confidentiality.

### Data transcription, translation, and analyses

After recording, the audio files were transferred to a computer and transcribed by an external expert. The transcripts were also subjected to quality control by listening to the recordings and rectifying any errors made. A thematic, inductive analysis based on Grounded Theory [32] was performed by two researchers. Firstly, the transcripts were attributed codes line by line following an inductive approach, creating emergent codes that grouped the content of each sentence or paragraph. The codes and groups were further examined for thematic patterns in the data. To facilitate the coding process, programs MAXQDA 2018 (VERBI GmbH, Berlin, Germany) and Atlas.ti 7.0 Scientific Software Development 2009 (GmbH, Berlin, Germany) were used. The researchers involved in the analysis performed the thematic analysis independently, subsequently combining their results, and discussing the data collection and analysis procedures in joint meetings. Triangulation of data sources and analyses was carried out to obtain concrete findings. Table 1 shows the categories and codes established in the analysis according to Tanahashi’s Health Service Coverage model.

**Table 1:** Categories and codes established in the analysis

**Accessibility**	Physical
	Administrative
	Financial
	Business hours
**Acceptability**	Knowledge of CD
	Stigma
	Willingness to use the service
**Contact**	Reason for seeking healthcare
**Effectiveness**	Health system response to user demand

## Results

Between 2015 and 2016, two FGSs, two TGSs, and fourteen individual interviews were conducted with women, and between 2017 and 2018, seven individual interviews were carried out with men (37 participants in total). Table 2 shows their sociodemographic characteristics.

**Table 2:** Sociodemographic data for the Bolivian population

**Code**	**Age**	**Region of origin**	**Level of education**	**Employment status**	**Public health coverage**	**Nº of children**	**Years in Spain**	**Pathway**
**WOMEN**								
**E1FP**	31	Cochabamba	Secondary education	Housekeeper	Yes	2	10	P/BD
**E2FP**	33	Santa Cruz	Primary education	Housekeeper	Yes	3	10	P/BD
**E3FP**	45	Cochabamba	Primary education	Housekeeper	Yes	4	9	NAST
**E4FP**	42	Vallegrande/ Santa Cruz	Primary education	Housekeeper	Yes	3	7	AST
**E5FP**	34	Cochabamba	Secondary education	Housekeeper	Yes	3	9	AST
**E6FP**	44	Santa Cruz	Secondary education	Beautician	Yes	1	13	AST
**E7FP**	31	Cochabamba	Secondary education	Housekeeper	Yes	2	10	NAST
**E8FP**	35	Santa Cruz	Primary education	Housekeeper	Yes	3	9	P/BD
**E10FP**	34	Cochabamba	Secondary education	Housekeeper	Yes	1	15	P/BD
**E11FP**	47	Cochabamba	Secondary education	Housekeeper	Yes	3	9	P/BD
**E12FP**	28	Santa Cruz	Secondary education	Housekeeper	Yes	1	6	AST
**E13FP**	47	Cochabamba	Secondary education	Housekeeper	Yes	3	9	P/BD
**E14FP**	50	Trinidad/ Santa Cruz	Secondary education	Unemployed	Yes	5	9	AST
**E15FP**	53	Santa Cruz	Secondary education	Cleaner	Yes	3	16	P/BD
**TG1FP**	31	Cochabamba	Secondary education	Housekeeper	Yes	2	10	NAST
	34	Cochabamba	Secondary education	Housekeeper	Yes	1	15	P/BD
	47	Cochabamba	Secondary education	Housekeeper	Yes	3	9	P/BD
**TG2FP**	28	Santa Cruz	Secondary education	Housekeeper	Yes	1	6	AST
	31	Cochabamba	Secondary education	Housekeeper	Yes	2	10	NAST
	31	Cochabamba	Secondary education	Unemployed	Yes	2	10	P/BD
**FG1FP**	44	Santa Cruz	Secondary education	Housekeeper	Yes	1	15	AST
	38	Santa Cruz	Primary education	Housekeeper	Yes	3	9	AST
	59	Santa Cruz	Secondary education	Unemployed	Yes	2	18	AST
	50	Trinidad	Secondary education	Housekeeper	Yes	5	14	P/BD
	46	Santa Cruz	Secondary education	Housekeeper	Yes	2	15	AST
	47	Santa Cruz	Secondary education	Housekeeper	Yes	3	16	AST
**FG2FP**	30	Santa Cruz	Secondary education	Community health agent	Yes	1	12	AST
	31	Cochabamba	Secondary education	Unemployed	Yes	1	9	AST
	42	Sucre	University education	Unemployed	Yes	3	10	AST
	56	Santa Cruz	Secondary education	Housekeeper	Yes	1	14	NAST
**MEN**								
**E16MP**	35	La Paz	Secondary education	Housekeeper	Yes	2	12	AST
**E17MP**	28	Cochabamba	University education	Civil servant	No (private coverage)	0	6	AST
**E18MP**	47	Tarija	Secondary education	Labourer and related trade worker	No	3	11	AST
**E19MP**	48	Cochabamba	Primary education	Labourer and related trade worker	Yes	3	15	AST
**E20MP**	47	Cochabamba	Primary education	Housekeeper	Yes	5	10	AST
**E22MP**	46	Cochabamba	Primary education	Labourer and related trade worker	Yes	3	25	AST
**E23MP**	52	Cochabamba	Secondary education	Labourer and related trade worker	Yes	3	18	AST

*FP* Female with positive result, *MP* Male with positive result, *P/BD* Pregnancy/Blood or organ donation, *AST* Actively seeking testing, *NAST* Not actively seeking testing

Most of the participants were women (81.1%), and 18.9% were men. The mean age of the respondents was 40.6 years old, ranging from 28 to 59. All participants had public health coverage with the exception of two men (5.4%). In terms of level of education, 78.4% of participants had completed secondary education or higher. The mean length of time they had been living in Spain was 11.6 years, and the majority reported having children (97.3%). 29.7% of the participants tested positive for CD following a blood donation or during pregnancy. While 13.5% did not actively seek a CD test, 56.7% of the participants (including all men interviewed) sought out a serological test for CD from healthcare services.

Based on Tanahashi’s Health Service Coverage model, interviewees contacted healthcare services for a CD test through three main pathways: 1) pregnancy or blood/organ donation; 2) an individual actively seeking CD testing, and 3) an individual not actively seeking CD testing.

### Pathway 1: Pregnancy and blood/organ donation

The women interviewed did not identify any physical, administrative, financial, or time-related accessibility problems in this pathway.

Regarding acceptability (the ability of the service to be accepted by the individual as per their social and cultural norms), the women interviewed reported that their reasons for using the service revolved around the health of their children and the altruistic act of donating blood. They explained that they wanted their children to grow up healthy and that donating blood was meant to do something good for someone else.

“For the baby’s sake… [to see] how [the baby] is progressing each month, whether it had [enough] vitamins or not… also for the mother’s sake.” E1FP

“I mean, I wanted to donate [blood] after a year of being here. […] I was happy, of course, to do [something] good for someone else as long as I [was able to]…” E11FP

In addition, the participants explained that they were satisfied with their pregnancy follow-ups as well as with their healthcare professionals; in fact, they emphasised the quality of the care they received as users and the good treatment they were given.

“Yes, when I was pregnant, I had all the tests done, they [the healthcare professionals] were always looking after me, they phoned me [to check on me] […] the nurses, the gynaecologist, everything went well.” E2FP

Moreover, the women interviewed indicated that their general knowledge of CD and about its transmission and symptoms was low prior to diagnosis, but their positive result created a need for them to increase their knowledge of and take an interest in CD.

“I said ‘What is that [CD]?’, because I hadn’t really heard about it before. ‘What is that? How does it affect me? What’s it going to feel like?’ Because if you don’t feel anything but they tell you that you have a disease… and it’s in your blood […] ‘How did I get infected?’ So many things crossed my mind…” E8FP

In terms of effectiveness (the ability of the service to satisfactorily meet health requirements), participants explained that they were diagnosed with CD at some point during pregnancy or postpartum. Women diagnosed through blood donation reported that after donation, they received a notification with their positive results along with advice to go to the hospital.

“When she was born […] they called me three days later and told me that I had to go back to the hospital because I’d been diagnosed… with Chagas disease.” E1FP

“They sent me a letter telling me I couldn’t be a blood donor because I had Chagas disease, and that I had to go to the hospital’s infectious diseases unit.” E13FP

Women also mentioned that healthcare professionals asked about their country of origin before requesting a serological test during pregnancy and postpartum follow-up.

“They asked me about my nationality […]. They said ‘Ah, then we’re going to request a Chagas test.’” E10FP

However, women also felt that their general practitioners (GPs) were not aware of the diagnosis, transmission, and treatment of CD; in fact, they pointed out that CD is better known among healthcare professionals who work in obstetrics and tropical medicine services.

“I don’t think GPs are aware of it […] most of them don’t know about that disease.” E1FP

Table 3 shows the barriers identified for pathway 1.

**Table 3:** Barriers identified in the pregnancy/blood donation pathway

**Accessibility**	No barriers identified
**Acceptability**	No barriers identified
**Effectiveness**	No barriers identified

### Pathway 2: an individual actively seeking CD testing

In terms of accessibility (physical, administrative, financial, etc.), participants reported having heard about the transmission and symptoms of CD and the possibility of being tested in Madrid from the people around them or their families, which is why they decided to seek testing.

“I heard about it on the radio and then, by word of mouth, I found out there was a campaign running. My friend had been there before.” E18MP

In addition, interviewees mentioned that they had sometimes encountered difficulties in accessing healthcare due to their status as irregular migrants, and that they did not receive medical care or were forced to pay for it as a result.

“They [the administrative staff] said ‘No, you have to legalise your status, otherwise you have to pay’.” E5FP

With respect to time-related accessibility, participants explained their difficulties in attending appointments, as their work schedules overlap with appointment slots and permission for time off to attend appointments is not always granted.

“If you have a job, your boss doesn’t really like it when you’re sick.” E14FP

In addition, a number of participants explained that healthcare facilities are too far away from their place of work or home, so it is not always possible for them to get tested for CD.

“I think there were 4 or 5 [healthcare] facilities, and all of them were far from her [his friend’s] home here in Madrid, so in the end she didn’t get tested.” E17MP

Regarding acceptability (the ability of the service to be accepted by the individual as per their social and cultural norms), interviewees explained that they decided to find out their CD status because their perceived risk was high enough. Several participants explained that they were born in an endemic region, while others had family members diagnosed with CD or who had died of CD. More specifically, the men interviewed explained that their decision to get tested was owed to their wives’ positive result during pregnancy or to the diagnosis or death of a family member.

“Then my wife said ‘We have Chagas, we have to go to the hospital!’, so we got tested […] my wife’s cousin’s heart stopped […] that’s when she was diagnosed with Chagas.” E19MP

With respect to effectiveness (the ability of the service to satisfactorily meet health requirements), interviewees reported that they obtained their diagnosis after getting an appointment with their GP.

“Then I asked my doctor and she told me that there was a chance (of being diagnosed). She sent me to another doctor […] at the hospital.” E4FP

However, other participants stated that, despite having reported their risk, their GP did not request any testing for them or refer them to another professional.

“I went to my healthcare facility, to my doctor, and said ‘See, my mother died of this [CD] and I’d like to know [my status] because sometimes I feel tired, agitated […] I think that’s not normal!’ […]. Then she said ‘OK’, she requested the [blood] tests, but the results… didn’t show that […]. Then she said ‘Maybe you have… a vitamin deficiency’.” E5FP

Table 4 shows the barriers identified for pathway 2.

**Table 4:** Barriers identified in people actively seeking CD testing

**Accessibility**	**Administrative:** Irregular migrant status.
	**Business hours:** their work schedules overlap with appointment slots.
	**Physical:** healthcare facilities are far away from their work place/home.
**Acceptability**	No barriers identified.
**Effectiveness**	**Health system response to user demand:** Lack of knowledge of the disease on the part of healthcare professionals.

Pathway 3: an individual not actively seeking CD testing

### Pathway 3: an individual not actively seeking CD testing

As in the second pathway, interviewees explained their difficulties in attending appointments due to their work schedules, physical distance, and working conditions.

“I think it’s because of lack of time […] because they [Bolivian people] work… you have to go to the doctor, get an appointment, and wait…” E19MP

“It took me an hour to get to the hospital […]. I have few [financial] resources now […] and I only work two hours [a day], and I know these things take time, but it’s just not possible for me […]. I had to change hospitals to one that is closer.” GF1

In addition, these participants also pointed out that their status as irregular migrants and the fact they have no health insurance card prevents them from accessing public healthcare or forces them to pay for the services provided.

“Yeah, if you don’t have a [health insurance] card, they don’t see you […]. Now it’s compulsory to have a work contract…” E3FP

Participants also explained that getting appointments with their GP made them feel embarrassed or worried because they think they might be disturbing them or making them feel uncomfortable.

“I didn’t go back to my doctor to ask for… another test, because I’m always bothering her [she laughs] ‘I want a blood test! A blood test!’” E7FP

With respect to acceptability (the ability of the service to be accepted by the individual as per their social and cultural norms), interviewees expressed doubts, lack of knowledge, and health-related beliefs about CD treatment and routes of transmission.

“Maybe it’s hereditary. But… it depends on where you live. But I don’t know… I don’t think so… Because my mum lived until she was almost 88 years old. If she’d had the disease, she would’ve died much earlier, wouldn’t she?” E23MP

Moreover, participants expressed that they only get an appointment with their GP when they have a health issue serious enough that it impacts their daily activities.

“Yeah, sometimes I feel ill, I feel tired and helpless, and [it’s only] then [when] I go to the doctor.” E3FP

Interviewees also reported that GPs are unfamiliar with the diagnosis, treatment, and symptoms of CD, and therefore feel that they are unable to help them.

“My GP didn’t know it [CD] existed [he laughs]. Right now, Spaniards haven’t heard of Chagas […]. I don’t think they know about that disease.” E16MP

Interviewees also explained that they were afraid to learn about their status, because they believed that being aware of a health problem meant accepting that they were ill, which would make their illness worse.

“For example, my son says ‘No, I don’t want to know […]. If I have it [CD], I’m going to worry’.” E20MP

“I have a friend that [says]… ‘I don’t go to the doctor because I don’t want to know [if I have CD]’.” E7FP

They also noted that the stigma associated with CD was also an issue when being diagnosed, as a positive test result is associated with poverty and living in rural settings.

“I think there must be people who may have Chagas and don’t want to get tested because they know they will test positive, because Chagas disease is associated with the poor […]. That can be a difficulty […] the shame, don’t you think?” E17MP

Regarding effectiveness (the ability of the service to satisfactorily meet health requirements), they explained that their physician decided to request a serological test for CD or referred them to another medical specialist. However, they also pointed out that getting tested was time-consuming, as they had to make several attempts or even consider other health problems before that.

“Then the haematologist said ‘I have a friend who is… in [the] tropical medicine [department] […]. Let’s try and see her, I’ll take you to her myself.’” E7FP

“Then I went to my GP and he said ‘You’re from Bolivia, you may have Chagas disease’.” E1FP

Table 5 shows the barriers identified for pathway 3.

**Table 5:** Barriers identified in people not actively seeking CD testing

**Accessibility**	**Administrative:** Irregular migrant status.
	**Business hours:** their work schedules overlap with appointment slots.
	**Physical:** healthcare facilities are far away from their work place/home
	**Professional:** psychological discomfort or embarrassment.
**Acceptability**	**Willingness to use the service:** Severity of symptoms, lack of trust in healthcare professionals, fear of knowing their CD status.
	**Stigma**
	**Knowledge of CD:** Knowledge and beliefs about transmission routes and treatment of CD.
**Effectiveness**	**Health system response to user demand:** Knowledge and training of healthcare professionals.

## Discussion

This study expands on the application of Tanahashi’s Health Service Coverage to CD by identifying the main CD diagnostic pathways: pregnancy and blood/organ donation, an individual actively seeking CD testing, and an individual not actively seeking CD testing.

**Fig. 2:**
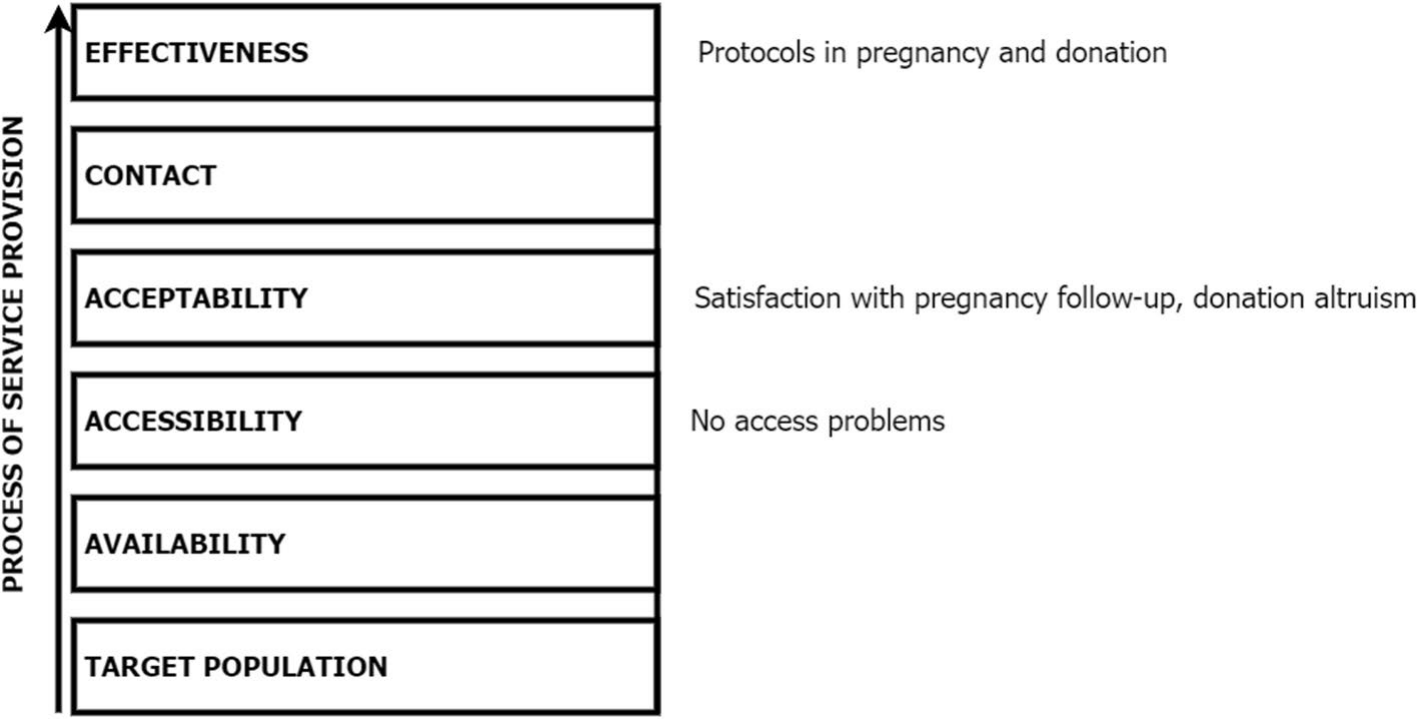
Diagram illustrating the effective coverage of the pregnancy or blood/organ donation pathway

Pregnant women and blood donors are part of the pathway with the least restrictive coverage (Fig. 2). CD testing is not widely offered, and this population uses health services for reasons not directly linked to CD, such as the altruistic act of donating blood or the maternal desire to monitor her pregnancy and the later birth of her child [33].

Spanish legislation in 2012 allowed medical care to be provided during pregnancy, delivery, and postpartum. As a result, no restrictions on public healthcare apply [25]. In addition, organ and blood donation requires serological testing for CD for persons born in endemic areas, persons whose mothers were born in endemic areas, and persons who have received a blood transfusion in an endemic country [34]. Protocols for testing pregnant women and donors are developed and available in some European countries, and a number of Spanish regions conduct routine CD screening, i.e., not based on a voluntary decision by the patient or the individual initiative of a health professional [7, 12, 14, 15]. As a result, most people matching the aforementioned profiles will be tested effectively.

**Fig. 3:**
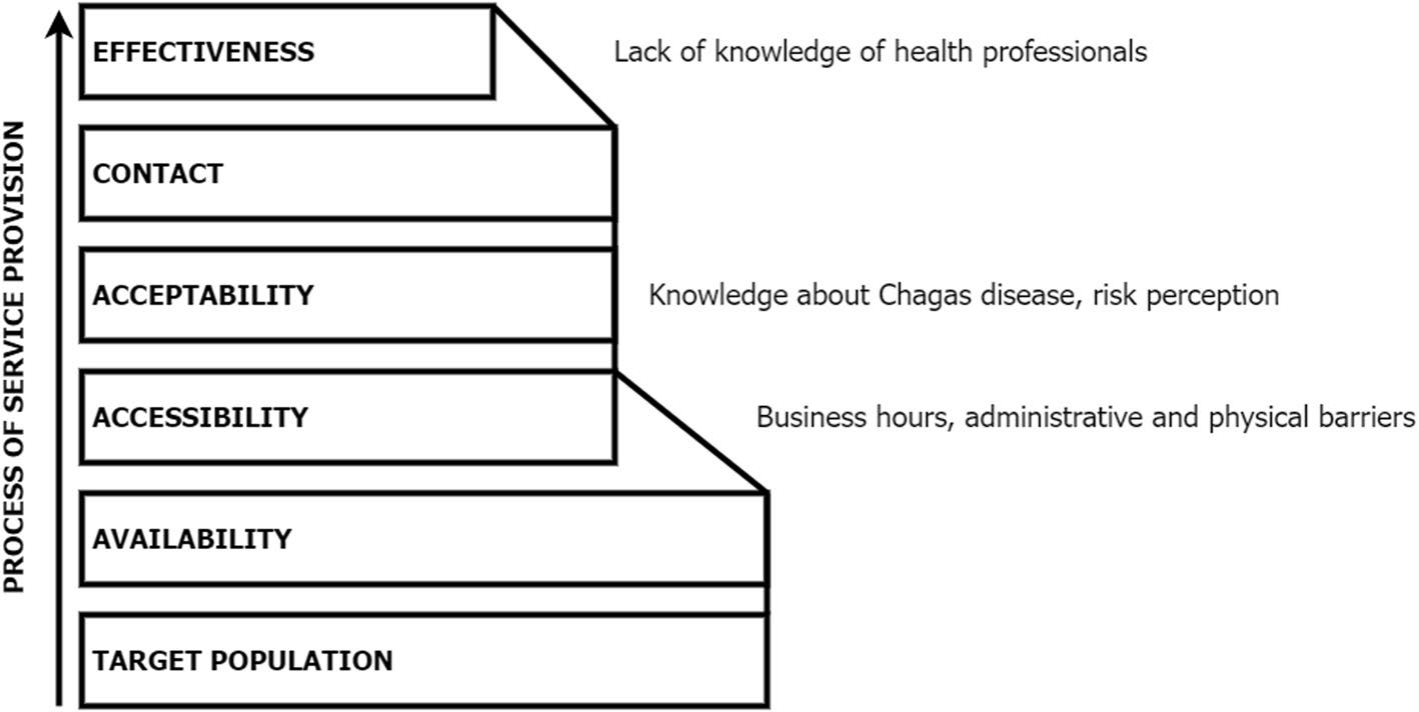
Diagram illustrating the effective coverage of individuals actively seeking CD testing

Regarding the second pathway, the individual who feels at risk and possesses knowledge about CD decides to make an appointment, which means that acceptability is high (Fig. 3). These factors have been described as determinants for making the decision to seek out testing [33, 35].

However, people on this pathway must overcome physical, administrative, and time-related constraints to access healthcare, such as legislation regarding access to the public health system, which is often decisive [26]. Their success will positively or negatively influence CD testing. A number of Spanish regions have developed community-based interventions for diagnosis and treatment to help with these difficulties [36, 37]. It is also well-known that there is a lack of knowledge about CD among GPs and other healthcare professionals, which means that effectiveness also depends on negotiation between the physician and the patient; the physician can either refer the patient to the hospital or request a test for them [38]. Therefore, the main bottlenecks in this pathway are accessibility and effectiveness.

**Fig. 4:**
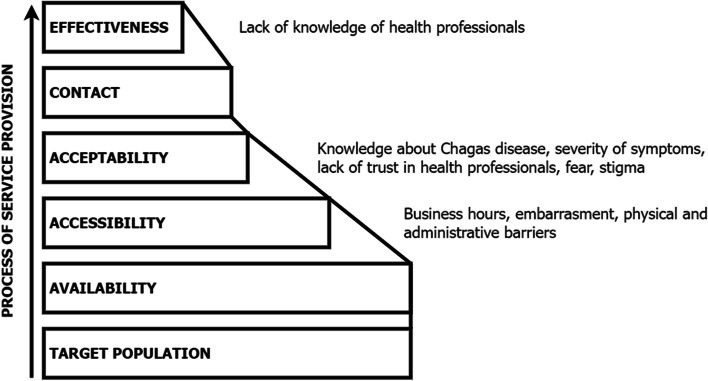
Diagram illustrating the effective coverage of individuals not actively seeking CD testing

Regarding the third pathway, the main factor that negatively influences CD diagnosis is acceptability: interviewees do not seek serological testing for CD due to their lack of knowledge about CD and a low perceived risk (Fig. 4) [33, 35].

Given that their working hours and administrative barriers are sufficiently restrictive, their use of health services depends on the severity of their illness (until symptoms interfere with their daily life). Stigma also affects acceptability because testing positive for CD still means death, misery, rejection, and social isolation despite the normalisation of CD among Bolivians [39–43]. This places the physician in a situation of diagnostic accuracy, because depending on his or her knowledge, the patient will either be diagnosed with CD or they will not. This pathway is definitely the one with the least effective coverage, as there are bottlenecks in terms of accessibility, acceptability, and effectiveness.

Consideration of these three pathways would seem reasonable to promote strategies that can influence acceptability and effectiveness. Screening protocols and specific training of healthcare professionals using these guidelines would establish working methods that would prevent patients making individual clinical decisions on their own [10, 38]. In addition, health education and counselling campaigns with the Bolivian population can increase acceptability by improving knowledge and enhancing risk perception [36, 37, 44–46].

Finally, public institutions must make an effort to remove barriers to access to healthcare and strive for universal health coverage [47].

The present study has some limitations. These findings are not necessarily applicable to other contexts because the study was conducted in Madrid, although its results could contribute to healthcare delivery for CD in non-endemic countries. In addition, our sample consists of Bolivian individuals diagnosed with CD who have resorted to the health system and shared their experiences and those of the people around them; however, there may be other individuals who do not reach out to the health system and whose accounts could provide additional, valuable information. Finally, it is important to highlight the reluctance of men to participate in the interviews and FGSs. This reluctance could be influenced by their ideals of masculinity, because seeking help or sharing problems is associated with weakness; these ideals could explain their absence in focus group sessions and their poor participation in interviews [39, 48].

## Conclusions

The most important pathways for CD diagnosis are pregnancy/blood donation, active search, and non-active search. Existing bottlenecks are less present during pregnancy and blood donation, and are greatest for individuals not actively and voluntarily seeking serological testing. Future screening protocols will need to take these bottlenecks into consideration in order to achieve effective coverage.

## Data Availability

The data that support the findings of this study are available from Repisalud repository (https://repisalud.isciii.es/), but restrictions apply to the availability of these data, which are not publicly available. Data are however available from the corresponding author upon reasonable request. Moreover, the protocols generated during the current study are available at Figshare (https://figshare.com/articles/dataset/Untitled_Item/14226710/2).

## Abbreviations

CD: Chagas disease
GP: General practitioners
